# Sutureless Intrascleral Haptic-Hook Lens Implantation Using 25-Gauge Trocars

**DOI:** 10.1155/2018/9250425

**Published:** 2018-12-27

**Authors:** Zhi-Xiang Hu, HaiShuang Lin, Lingying Ye, Zhong Lin, Tianyu Chen, Ke Lin, Rong-Han Wu

**Affiliations:** Eye Hospital, School of Ophthalmology and Optometry, Wenzhou Medical University, 270 Xueyuan Road, Wenzhou, Zhejiang Province 325027, China

## Abstract

**Purpose:**

To report a new technique for sutureless intrascleral fixation of three-piece foldable intraocular lenses (IOLs) using 25-gauge trocars.

**Methods:**

We performed this technique on patients with insufficient posterior capsule support. Seventeen eyes from 15 patients with aphakia, dislocated IOL, or subluxated crystalline lens undergoing posterior chamber sutureless implantation of an IOL were studied. The haptics of the IOL were externalized using two 25-gauge forceps. The haptics were bended back (hook-like) into the vitreous cavity through a scleral incision made by using a 25-gauge trocar. And, IOL tilt was determined by using a slit lamp and UBM, and complications were recorded.

**Results:**

The IOLs were fixed with exact centration and axial stability. No wound leakage was reported even without the use of sutures. The mean best-corrected visual acuity (BCVA) was 0.82 logarithm of the minimum angle of resolution (logMAR) units preoperatively, and the mean BCVA was 0.44 logMAR units at the 6-month follow-up visit. No postoperative retinal detachment, endophthalmitis, IOL tilt or dislocation, or vitreous hemorrhage was noted.

**Conclusion:**

Sutureless intrascleral haptic-hook posterior chamber IOL implantation using 25-gauge trocars provides good IOL fixation with reliable wound closure without the use of sutures. This trial is registered with ChiCTR1800017436.

## 1. Introduction

Intrascleral fixation of posterior chamber (PC) intraocular lens (IOL) has become more popular, as it has advantages such as minimal trauma to the surrounding tissues, good IOL stabilization decreasing the incidence of IOL tilt along with shorter operation time, and does not require degradable threads which may lead to long-term extraconjunctival exposure [1–5]. The various intrascleral fixation techniques have critical differences in the manner in which the haptic of the IOL is handled [6]. Agarwal et al. and Oh et al. have reported the use of fibrin glue-assisted sutureless IOL scleral fixation combined with scleral flaps for the implantation of PC-IOLs [7, 8]. Takayama et al. have described a modified technique where the IOL haptics are incarcerated into the prepared scleral tunnels instead of a scleral flap, which provides stability for the PC-IOLs [9]. Ohta et al. created a Y-shaped scleral incision to fix the haptic and improved wound closure [10].

We modified the haptic fixation to increase the stability of the IOL haptic by bending the haptics back into the vitreous cavity by 25-gauge vitrectomy to minimize the scleral incisions. And, we performed this modified technique in a series of eyes with aphakia, dislocated IOL, or subluxated crystalline lens. In this article, we report a case series of this technique and its clinical results.

## 2. Materials and Methods

### 2.1. Subjects

*Retrospective Study*. The data of patients with aphakia, dislocated IOL, or subluxated crystalline lens who underwent posterior chamber sutureless implantation of an IOL at the Eye Hospital of Wenzhou Medical University between April 2017 and December 2017 were collected. An informed consent was obtained from all participants. All study procedures adhered to the tenets of the Declaration of Helsinki. All of the patients underwent ophthalmologic examination including measurements of BCVA, slit-lamp examination, and measurement of intraocular pressure (IOP) at preoperative and postoperative visits. Ultrasound biomicroscope (UBM) examination was performed at follow-up to evaluate the centration and axial stability of the IOL, and complications were recorded.

### 2.2. Surgical Procedure

The surgery was performed under retrobulbar anesthesia. The conjunctiva is cut open for 3.0 mm at two points 180° apart, usually at 4 o'clock and 10 o'clock positions, and light scleral cautery is used to achieve adequate hemostasis. Whole or partial vitrectomy will be needed for dislocated IOL or subluxated crystalline lens.

**Figure 1:**
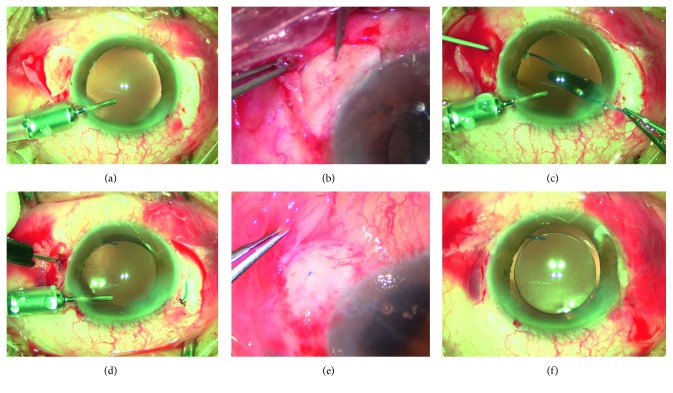
Photographs showing the haptic-hook lens implantation. (a) Sclerotomy 2.0 mm from the limbus previously created for 25-gauge vitrectomy. (b) Creation of the scleral groove with paracentesis blade. (c) Externalization of the leading haptic with 25-gauge forceps. (d) The haptic is bent and inserted back into the vitreous cavity through the second scleral incision created by a 25-gauge trocar blade. (e) Hide of the haptics to the intrascleral groove. (f) Conjunctival incisions are closed with 8-0 absorbable suture.

Two 25-gauge cannulas are placed exactly 180° apart at 4 o'clock and 10 o'clock and are perpendicularly inserted through the sclera, and pars plana 1.5–2.0 mm away from the limbus (Figure 1(a)), usually 2.0 mm when combined with vitrectomy. This precise positioning is required to ensure good lens centration. This is followed by 25-gauge anterior vitrectomy to remove the prolapsing vitreous in the anterior chamber or the whole vitreous through the cannulas if needed. Subsequently, the cannulas at two points 180° apart are removed, and the 25-gauge trocar blade is used to create 2 ciliary sulcus-based scleral incisions of about 1 mm away from the former scleral incisions at two points 180° apart. These scleral incisions are parallel to the limbus and must be made in a uniplanar manner. Intrasceral grooves are made between the two adjacent scleral incisions (Figure 1(b)).

A superior 3.0 mm clear main corneal incision and auxiliary corneal incision are made which are used for injection of a 3-piece PC-IOL (Matrix Acrylic Aurium 400; Medennium Inc.; California, USA) into the anterior chamber. The 25-g forceps are passed through the trocar incision at the 4 o'clock position, and the leading haptic is grasped and extracted from the eye (Figure 1(c)). The IOL with the trailing haptic is inserted into the anterior chamber. The trailing haptic is also held with forceps, and both haptics are externalized onto the sclera. The haptics are bent and inserted back into the vitreous cavity through the adjacent scleral incision (Figure 1(d)) while the outer parts of the haptics are perfectly buried in the intrascleral groove (Figure 1(e)). The smooth implementation of the above steps benefits from the flexible haptics of Matrix Acrylic Aurium 400. The material of this haptics is polyvinylidene fluoride (PVDF), the configuration is modified-C, and the angulation is 5 degrees. All above make bending back the haptics into the vitreous cavity easy. After the closure of the clear main corneal incision with hydration, the conjunctival incisions are closed with 8-0 absorbable sutures (Figure 1(f)).

**Figure 2:**
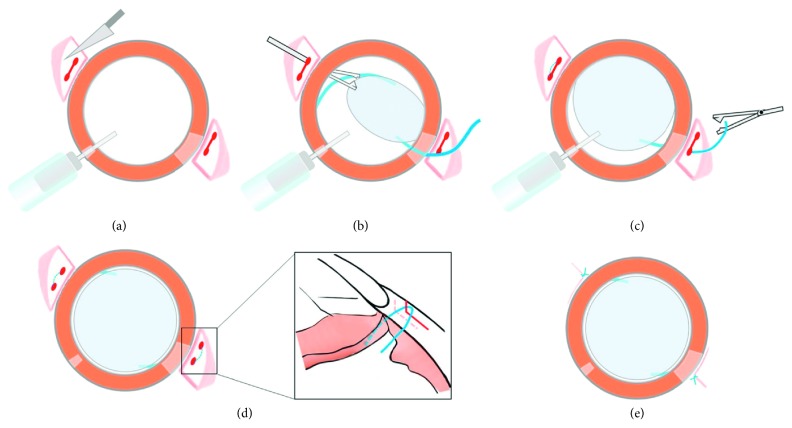
Diagram of the surgical procedure.

Diagrams of the key steps of the procedure are shown in Figure 2.

## 3. Results and Discussion

**Figure 3:**
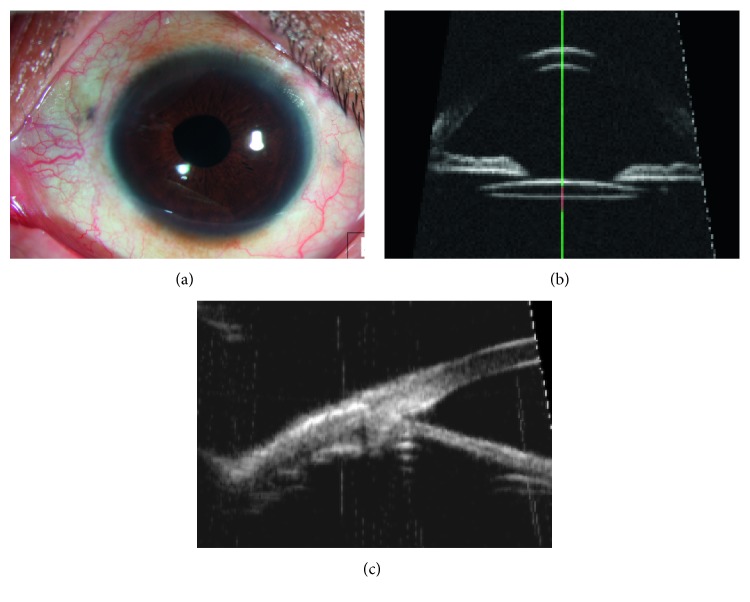
Six months postoperative anterior segment examinations. (a) Slit-lamp microscopy showing the scleral wounds. (b) UBM image showing the centration of the lens. (c) UBM image showing the IOL haptic position in the sclera.

Our modified sutureless technique was performed in 15 patients. The technique was combined with vitrectomy to partially or entirely remove the vitreous in all patients. The mean BCVA was 0.82 logMAR units preoperatively and 0.44 logMAR units at the 6-month visit (Table 1). Patients who came back for follow-up showed good lens centration and stable haptic fixation (Figure 3). Complications such as postoperative inflammation, hyphema, decentration, glaucoma, corneal edema, or wound leakage have not been observed in the 6-month follow-up examinations (Table 2). None of the eyes has required subsequent surgical procedures.

Intrascleral IOL fixation has frequently been used in eyes with inadequate posterior capsular or zonular support [11], as this technique has advantages over conventional transscleral suturing of the IOL. However, stability of the haptic of the IOL has been reported to be the area of most concern in long-term visits [12]. At the same time, the surgeon must be aware of the thickness of the sclera and perform the technique cautiously, especially in vitrectomized eyes regardless of the use of scleral flaps or tunnels for haptic fixation.

We found that scleral tunnels used to hide the haptic might result in several complications, such as slippage of the haptic and intraocular lens pupillary capture. These complications may occur due to high strain of the IOL and a flabby iris over the long term. Yamane et al. [12] have reported similar complications and used a flange to prevent haptic dislocation. In contrast, we used a hook-like fixation assisted by 25-gauge trocars to completely prevent slippage of the greater part of the haptic. And, we could reposition the lens through the flexible haptic bending according to various distances between two incisions, which may be irretrievable in flanged haptic. Because we bury the haptic intrasclerally, patients have less conjunctival irritation compared with exposed flanged haptic.

There are many advantages to our modified technique. First, we reduced the number of scleral incisions to minimize wounds when combined with vitrectomy and avoid ocular hypotension after surgery. Multiple incisions may increase outflow of the vitreous humor, especially in vitrectomized and pathologic eyes with myopia. And scleral incision sutures will be necessary under these conditions. A 26-gauge or smaller syringe needle could be used instead of a 25-gauge trocar in noncombined vitrectomy eyes. Second, the haptics are inserted back into the vitreous cavity, which prevent slippage of the haptic, decrease incidence of IOL tilt, and increase stability of IOL. Third, the outer parts of the haptics are perfectly buried in the scleral groove to lower the risk where the IOL haptics are exposed below conjunctiva and get infected. In addition, it is unlikely to damage the haptics of the 3-piece PC-IOL (Matrix Acrylic Aurium 400) when bending the haptics for its flexibility and extensivity. The modified technique does not require degradable threads which may lead to long-term extraconjunctival exposure and simplify the procedure with shorter operation time.

The limitation of the study is the number of cases is few and the period of follow-up is too short to evaluate the outcome. And, we judge the IOL tilt by using a slit lamp and UBM instead of optical coherence tomography images so that we cannot evaluate the specific IOL tilt. Long-term follow-up and prospective, interventional study are necessary to analyze the anatomic and functional recovery.

## 4. Conclusions

Our modified haptic-hook technique for intrascleral fixation of PC-IOLs without the use of scleral flaps might be useful for IOL implantation in eyes without sufficient capsule support. We believe that this technique is better than those previously reported because the number of the sclerotomy is less and the haptics are inserted back into the vitreous cavity. In addition, this operation is easy to carry out. Nevertheless, long-term anatomic and functional recovery in patients who have undergone surgery using this technique merit further study.

## Figures and Tables

**Table 1:** Baseline characteristics and postoperative data of the patients.

Characteristics	Data
Number of eyes (patients)	17 (15)
Age, years	56.4 ± 13.5
Male/female, *n*	7/8
Baseline logMAR BCVA	0.82 ± 0.89
logMAR BCVA at 6 months^a^	0.44 ± 0.45
Baseline IOP, mmHg	18.0 ± 3.0
IOP at 1 week, mmHg^a^	14.8 ± 7.2
IOP at 6 months, mmHg^a^	17.1 ± 2.9

All values stand for mean ± standard deviation. BCVA = best-corrected visual acuity; IOP = intraocular pressure. ^a^Compared by Fisher's exact test; others compared by two-paired test.

**Table 2:** Clinical features and postoperative surgical outcome of each eyes.

Cases	Gender/age	Operated eye	Associated ocular conditions from previous surgery	Type of dislocation at presentation	Preoperative logMAR BCVA	Postoperative logMAR BCVA at 6 months	Postoperative complications
1	M/53	Right	PCR and sulcus-fixated IOL	Out-of-the-bag	0.22	0.50	None
2	M/46	Right	PCR and sulcus-fixated IOL	In-the-bag	1.30	1.30	None
3	M/24	Left	PCR and sulcus-fixated IOL	Out-of-the-bag	0.22	0.22	None
4	M/48	Right	PCR and sulcus-fixated IOL	Out-of-the-bag	0.05	0	None
5	M/48	Left	Marchesani syndrome, subluxated crystalline lens	In-the-bag	0.10	0	None
6	M/75	Left	Trauma, aphakic	Absence of capsular bag	2.60	1.30	None
7	M/58	Left	Trauma, aphakic	Absence of capsular bag	0.10	0.22	None
8	F/53	Left	Trauma, luxated crystalline lens	Out-of-the-bag	0.40	0.52	None
9	F/59	Right	PCR and sulcus-fixated IOL	Out-of-the-bag	2.30	0.40	None
10	F/59	Left	PCR and sulcus-fixated IOL	Out-of-the-bag	2.60	0.22	None
11	F/69	Right	PCR and sulcus-fixated IOL	Out-of-the-bag	0.70	0.70	None
12	M/72	Left	Subluxated crystalline lens	In-the-bag	1.00	0.10	None
13	F/35	Right	Subluxated crystalline lens	In-the-bag	0.40	0.40	None
14	F/60	Right	Luxated crystalline lens	In-the-bag	0.10	0.15	None
15	F/68	Right	Subluxated crystalline lens	In-the-bag	1.00	0.52	None
16	F/60	Left	Subluxated crystalline lens	In-the-bag	0.00	0.00	None
17	F/71	Right	Luxated crystalline lens	In-the-bag	0.80	1.30	None

BCVA = best-corrected visual acuity; PCR = posterior capsule rupture.

## Data Availability

All data generated or analyzed during this study are included in this article.

## References

[1] Vote B. J., Tranos P., Bunce C., Charteris D. G., Da Cruz L. (2006). Long-term outcome of combined pars plana vitrectomy and scleral fixated sutured posterior chamber intraocular lens implantation. *American Journal of Ophthalmology*.

[2] Burcu A., Yalniz-Akkaya Z., Abay I., Akif Acar M., Ornek F. (2014). Scleral-fixated posterior chamber intraocular lens implantation in pediatric and adult patients. *Seminars in Ophthalmology*.

[3] Karadag R., Bayramlar H., Cakici O. (2015). Sutureless scleral fixation of intraocular lenses. *Graefe’s Archive for Clinical and Experimental Ophthalmology*.

[4] Haszcz D., Nowomiejska K., Oleszczuk A. (2016). Visual outcomes of posterior chamber intraocular lens intrascleral fixation in the setting of postoperative and posttraumatic aphakia. *BMC Ophthalmology*.

[5] Totan Y., Karadag R. (2012). Trocar-assisted sutureless intrascleral posterior chamber foldable intra-ocular lens fixation. *Eye (Lond)*.

[6] Karadag R., Celik H. U., Bayramlar H., Rapuano C. J. (2016). Sutureless intrascleral fixated intraocular lens implantation. *Journal of Refractive Surgery*.

[7] Agarwal A., Kumar D. A., Jacob S., Baid C., Agarwal A., Srinivasan S. (2008). Fibrin glue-assisted sutureless posterior chamber intraocular lens implantation in eyes with deficient posterior capsules. *Journal of Cataract and Refractive Surgery*.

[8] Oh S. Y., Lee S. J., Park J. M. (2015). Comparision of surgical outcomes of intraocular lens refixation and intraocular lens exchange with perfluorocarbon liquid and fibrin glue-assisted sutureless scleral fixation. *Eye (Lond)*.

[9] Takayama K., Akimoto M., Taguchi H. (2015). Transconjunctival sutureless intrascleral intraocular lens fixation using intrascleral tunnels guided with catheter and 30-gauge needles. *British Journal of Ophthalmology*.

[10] Ohta T., Toshida H., Murakami A. (2014). Simplified and safe method of sutureless intrascleral posterior chamber intraocular lens fixation: Y-fixation technique. *Journal of Cataract and Refractive Surgery*.

[11] Cho Y. W., Chung I. Y., Yoo J. M. (2014). Sutureless intrascleral pocket technique of transscleral fixation of intraocular lens in previous vitrectomized eyes. *Korean Journal of Ophthalmology*.

[12] Yamane S., Sato S., Maruyama-Inoue M., Kadonosono K. (2017). Flanged intrascleral intraocular lens fixation with double-needle technique. *Ophthalmology*.

